# When the Appendix Takes a Detour: A Rare Case of the Appendix Intussuscepting Into the Caecum Mimicking an Appendiceal Mucocele

**DOI:** 10.7759/cureus.80965

**Published:** 2025-03-21

**Authors:** Ming Chuen Chong, Neil Wu, Abdelhamed Fanta, Osama Shakil, Nasir Z Ahmad

**Affiliations:** 1 Colorectal Surgery, University Hospital Limerick, Limerick, IRL

**Keywords:** adult intussusception, appendiceal endometriosis, appendiceal intussusception, appendiceal mucocele mimic, gastrointestinal endometriosis, laparoscopic caecectomy, minimally invasive surgery, rare appendiceal pathology

## Abstract

Acute appendicitis is a common condition, whereby accurate diagnosis relies on multiple modalities in conjunction with a thorough history and clinical examination. Imaging, particularly computed tomography (CT), is highly sensitive in reliably determining the position of the appendix. It is not unusual for appendicitis to present in atypical ways or be masked as other intra-abdominal conditions, complicating the diagnosis. This report discusses a rare case of intussusception of the appendix into the caecum, with a histopathological specimen later proving the intraoperative specimen as endometrial tissue, highlighting an uncommon presentation. We report the case of a 51-year-old woman who experienced worsening abdominal pain, nausea, and early satiety over several months. Initial evaluation revealed microcytic anaemia and a smoking history of 25 pack-years. Imaging strongly suggested an appendiceal mucocele. A colonoscopy revealed a large caecal polyp originating from within the appendiceal orifice. A multidisciplinary team discussion recommended performing a diagnostic laparoscopy to further guide management. While conducting the laparoscopic surgery, a lump on the taenia coli obscured the view of the appendix, necessitating a partial caecectomy. Postoperative examination of the specimen confirmed intussusception of the appendix into the caecum, later verified by a pathologist to be appendiceal endometriosis. This case highlights the significant diagnostic challenges linked to adult intussusception. Although CT scans and colonoscopy are vital in preoperative assessments, correctly identifying the underlying condition can be difficult. The laparoscopic approach has shown both safety and effectiveness in diagnosis and treatment. Prompt surgical intervention is crucial for resolving intussusception and treating appendicitis to prevent serious complications.

## Introduction

Acute appendicitis has an incidence of 100 to 223 new cases per 100,000 individuals annually [1]. Appendicitis involves an appendiceal luminal obstruction that causes a rise in intraluminal and intramural pressure resulting in an inflammatory process [2]. The appendix becomes mucus-filled and colonised with bacteria, causing it to become susceptible to perforation. In the case of acute appendicitis, the outer diameter threshold of the appendix is 6 mm, with >15 mm indicating the presence of a mucocele [3]. Intussusception is a serious condition whereby a part of the intestine folds into the section adjacent to it, commonly involving the small bowel [4]. Symptoms are quite nonspecific, involving abdominal pain, vomiting, bloating and bloody stool. The usual age of occurrence is 6 to 18 months old [5]. Hence, this is a rare occurrence in adults, accounting for 5% of intussusception cases [6]. In exceptionally rare cases, mucocele of the appendix may present as intussusception with accompanying endometriosis.

Endometriosis is a fairly common chronic condition, whereby functional tissue lining the uterus (endometrial glands and stroma) implants outside of the uterine cavity [7]. Gastrointestinal endometriosis (GE) occurs in 3-37% of all endometriosis cases, while appendiceal endometriosis represents approximately 3% of GE cases and less than 1% of all endometriosis cases [8]. Here, we illustrate a rare case of appendiceal endometriosis that appeared to be intussusception intra-operatively, evolving from what appeared to be an appendiceal mucocele on imaging pre-operatively.

This article was previously presented as a poster at the 2025 Sylvester O'Halloran Perioperative Symposium on February 28, 2025.

## Case presentation

The patient is a 51-year-old perimenopausal woman who was referred to the emergency department by her General Practitioner (GP) with worsening epigastric pain, nausea and early satiety. These symptoms had been persistent for the past few months, but the worsening pain prompted her to visit the GP. The patient described a burning pain in the epigastric region, exacerbated by food ingestion and lying down. Over-the-counter analgesia did not relieve the pain. Associated symptoms included generalised fatigue. However, she did not report vomiting, hematemesis, melena or weight loss. She had no gynaecological history, and her last menstrual period was six months ago. Her menstrual periods had always been regular and adhered to a 30-day cycle. Her medical history was significant for microcytic hypochromic anaemia on routine testing and hypertension well-controlled with telmisartan 40mg OD. With regard to social history, she had a 25-pack-year smoking history, denied alcohol use, and did not engage in illicit substances. Her family history was unremarkable. Review of systems was insignificant.

On examination, the patient had a soft abdomen but a tender epigastric region on palpation. There were negative Rovsing’s sign, Psoas sign and Murphy’s sign. Bowel sounds were audible and normal. There were no stomas, hernias, or scars suggestive of previous surgeries. The cardiovascular and respiratory examinations were unremarkable. She was vitally stable in terms of blood pressure and respiratory rate and was apyrexial. Table 1 shows her blood results on the initial presentation.

**Table 1:** Blood laboratory results on the initial presentation showing iron-deficiency anaemia with normal B12 levels.

	Laboratory Results	Normal Values
Hb	9.6 g/dL	12 – 16 g/dl
Serum Iron	4 umol/L	9 – 30 umol/L
Transferrin	3.9 g/L	2.0 – 3.6 g/L
Transferrin Saturation (calculated)	4%	15 – 45 %
TIBC (calculated)	98 umol/L	41 – 77 umol/L
Ferritin	6.9 ng/mL	13 – 150 ng/mL
Vit B12	971 pg/mL	197 – 771pg/mL
CRP	1 mg/dL	0.8 – 1 mg/dL

The initial working diagnosis was gastritis. Hence, she was admitted under Gastroenterology and managed symptomatically with a proton-pump inhibitor, intravenous pantoprazole (40 mg twice daily).

**Figure 1:**
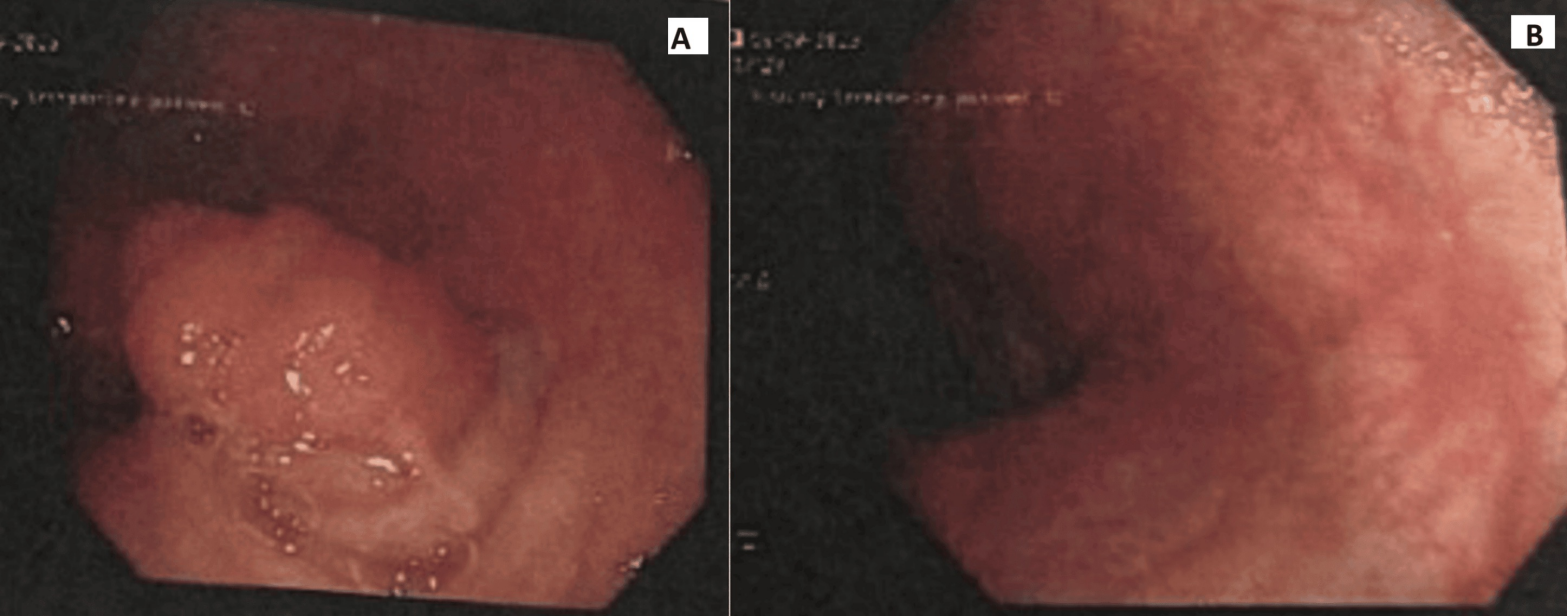
Oesophagogastro duodenoscopy (OGD) showing gastritis, no evidence of metaplasia, atrophy, dysplasia, or malignancy.

**Figure 2:**
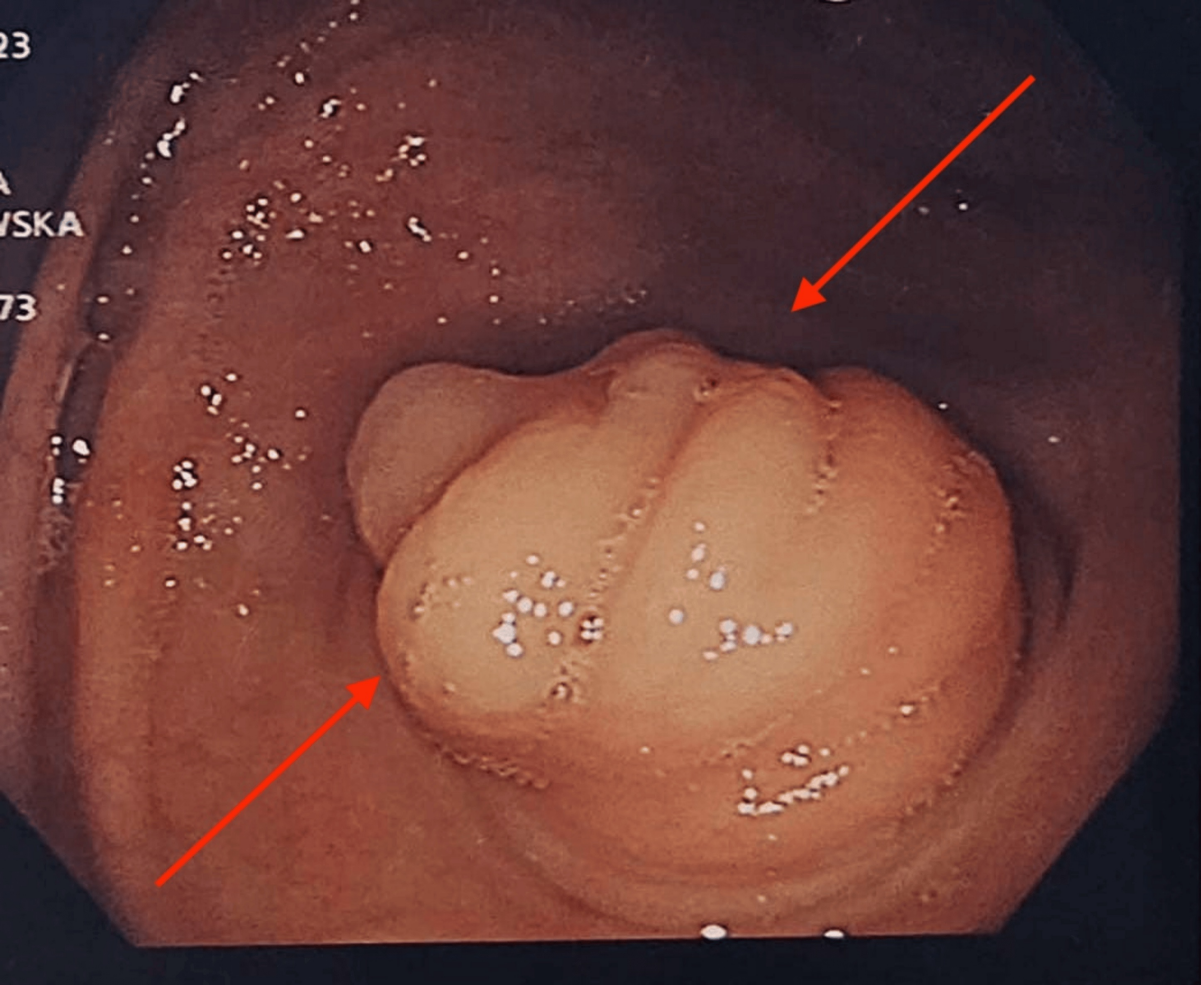
Polypoid mass in the caecum on colonoscopy. Biopsy results showed a tubular adenoma of the large bowel with low-grade dysplasia and no invasion.

**Figure 3:**
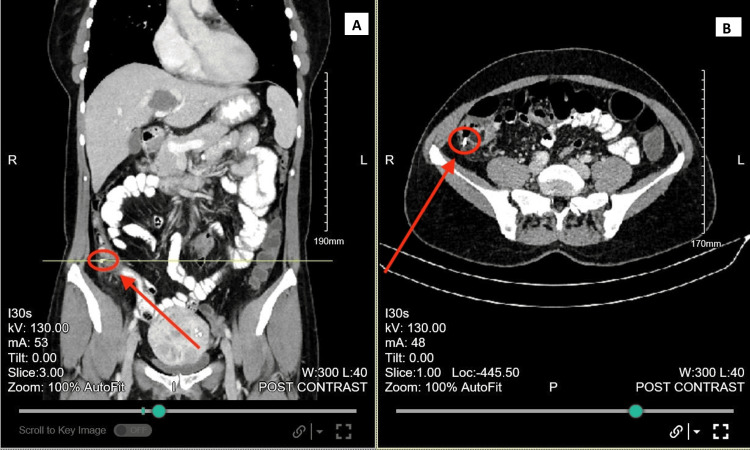
Computed tomography (CT) scan showing an appendiceal mucocele (coronal view on left and axial view on right).

An elective oesophagogastro duodenoscopy (OGD) and biopsy were performed (Figure 1). From the antral biopsy, there was acute and chronic Helicobacter pylori-associated gastritis within gastric antral-type mucosa, but no evidence of metaplasia, atrophy, dysplasia, or malignancy. An elective colonoscopy was performed two months later, which showed a 2 cm polyploid appendicular mass in the caecum (Figure 2). Biopsy samples were obtained and showed a tubular adenoma of the large bowel with low-grade dysplasia and no invasion. The ascending colon polyp showed a sessile serrated lesion of the large bowel. The patient was then referred to Colorectal Surgery for surgical intervention of the appendiceal mucocele. A CT scan showed a nodule adjacent to the distal cecal pole representing an appendiceal mucocele (Figure 3).

With the above investigations completed, a multidisciplinary meeting was conducted involving the gastroenterology medical team and colorectal surgical team. It was decided that the best management plan would be for the patient to undergo surgical resection of the appendiceal mucocele with an elective laparoscopic appendectomy and partial caecectomy.

**Figure 4:**
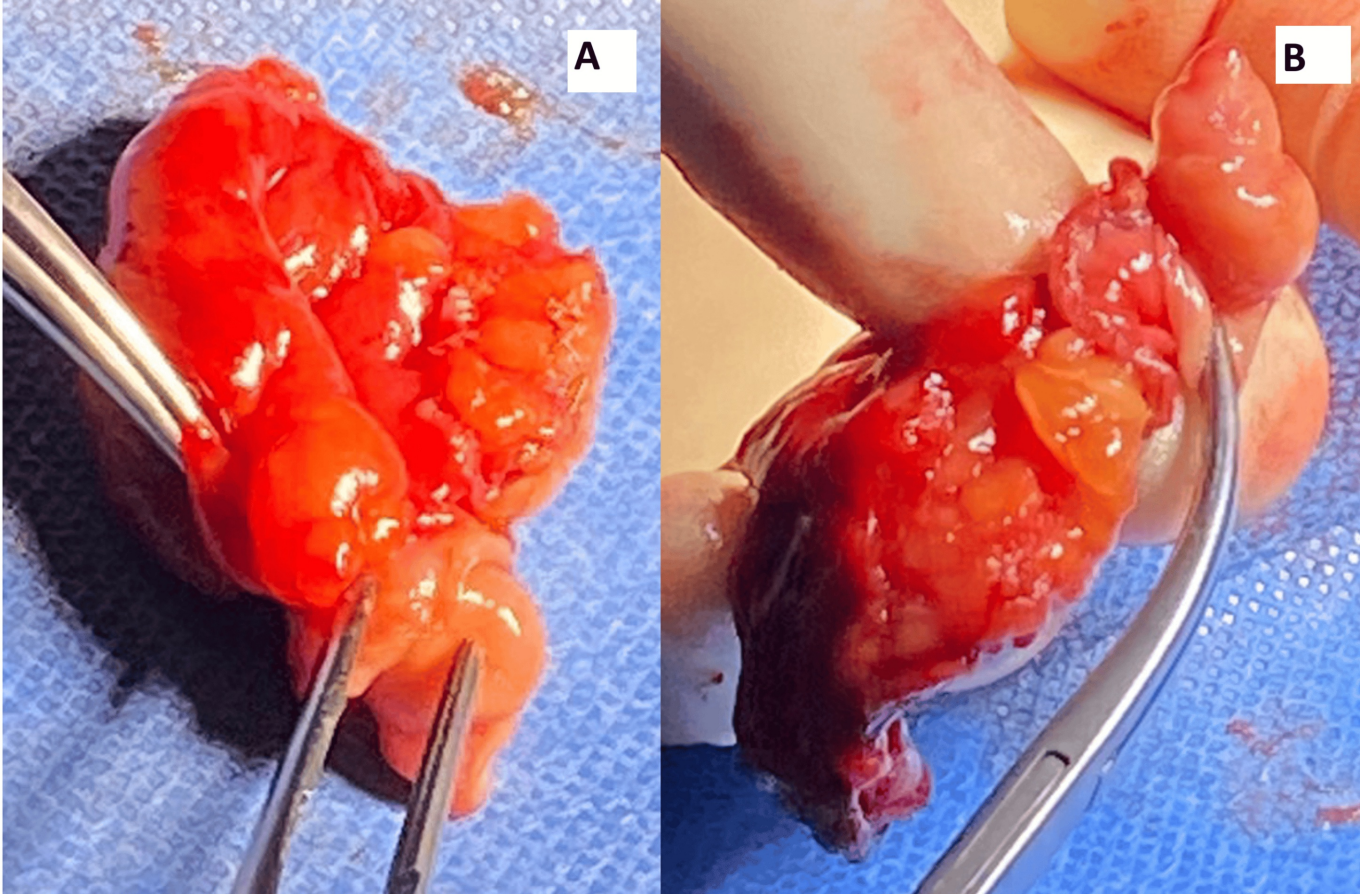
Surgical specimen of the appendix, whereby there was prolapse of the proximal bowel along with its mesenteric fold into the lumen of the adjacent distal bowel, indicative of intussusception.

A laparoscopic approach was utilised for the procedure. Three ports were inserted as follows: an infra-umbilical 11mm port for primary access, a left quadrant 10mm port used for pneumoperitoneum and as the camera port, which was later converted to 12mm, and a supra-umbilical 5mm port. On initial inspection, a lump was noted along the taenia coli of the cecum, which made it difficult to visualise the appendix. Due to this, identifying the position of the appendix proved technically challenging. To improve access and visibility, thorough lateral mobilisation of the right colon was performed. Despite these efforts, the appendix could not be located. Attention was then directed to the caecal lump as the likely pathological source. The lump was carefully dissected, and a stapler was used to perform a partial caecectomy, targeting the lump. The specimen was examined intra-operatively (Figure 4). On dissection, findings revealed prolapse of the proximal bowel along with its mesenteric fold into the lumen of the adjacent distal bowel, indicative of intussusception. The procedure was completed laparoscopically without complications. The patient was discharged day one post-operation and had a successful recovery. The specimen was sent for histopathological evaluation which revealed endometrial tissue, giving the final diagnosis of appendiceal endometriosis to be the cause of her symptoms. The patient was followed up in the surgical outpatient clinic three months later and showed good progress in recovery, where her initial symptoms on presentation had resolved completely.

## Discussion

The first account of endometriosis dates back to 1860 by Karl Freiherr von Rokitansky [9]. Endometriosis is a condition whereby there is growth of endometrial tissue outside of the uterine cavity. The most common endometriosis involvement is the ovaries (54.9%) and 3%-37% involves the gastrointestinal tract. Among those with gastrointestinal involvement, the appendix is the least commonly seen at 3%. Endometriosis can also be occasionally discovered in the umbilicus and surgical scars, especially following caesarean section [10,11].

Intussusception is defined as the invagination of one segment of the bowel into an immediately adjacent segment of the bowel, occurring mainly in the paediatric population. Idiopathic ileocaecal intussusception is the most common form in children, while intussusception in adults is uncommon and occurs more often in the small intestine than in the colon [12]. Adult intussusception is rare, occurring in 5% of all intussusception cases, presenting clinically as recurrent abdominal pain, bloody stool and abdominal pain [13].

The underlying pathophysiology of appendiceal endometriosis remains uncertain, but proposed mechanisms include retrograde menstruation, lymphatic spread, haematogenous spread, and coelomic metaplasia. These factors contribute to its varied clinical manifestations, ranging from asymptomatic cases to presentations mimicking acute appendicitis, intussusception, or even neoplastic conditions.

Diagnosing appendiceal endometriosis preoperatively is particularly challenging due to its nonspecific symptoms and overlapping features with other gastrointestinal and gynaecological conditions. In this case, the patient initially presented with epigastric pain, nausea and early satiety, symptoms more commonly associated with gastric pathology than appendiceal disease. Furthermore, endometriosis is oestrogen-dependent, which makes perimenopausal and postmenopausal endometriosis rare, due to the reduction or absence of oestrogen hormone production [14]. The presence of microcytic anaemia warranted further evaluation, as chronic gastrointestinal blood loss is a key concern. Despite undergoing a comprehensive diagnostic workup, including colonoscopy and CT imaging, the underlying pathology was initially misinterpreted as an appendiceal mucocele. This illustrates the inherent limitations of imaging modalities, as appendiceal endometriosis does not have distinctive radiological features and may be mistaken for other conditions, particularly in the absence of a classic history of endometriosis. This case highlights the indispensable role of histopathological examination in accurately diagnosing appendiceal endometriosis, particularly in rare presentations where imaging and clinical suspicion alone may be difficult to derive the diagnosis.

At present, there is no clear clinical guideline on the management of appendiceal endometriosis [15]. Surgical management remains the mainstay of treatment for symptomatic appendiceal endometriosis. Perhaps this could be attributed to the fact that diagnosis tends to be retrospective, postsurgical intervention, as seen in this case report and multiple others. In this case, the presence of a caecal lump during laparoscopy obscured the visualisation of the appendix, leading to the decision to perform a partial caecectomy rather than a standard appendectomy. A partial caecectomy should be performed if clear resection margins cannot be achieved because the mass lesion involves the caecum. This approach was utilised in a similar case report and review of literature, whereby a partial caecectomy was required when the base of the mucocele was broad and protruded into the cecal wall [16].

This underscores the importance of intraoperative adaptability, particularly when preoperative imaging findings do not correlate with intraoperative observations. The laparoscopic approach was particularly advantageous, offering both diagnostic and therapeutic benefits with minimal invasiveness. Laparoscopy allowed for thorough abdominal exploration, identification of the pathology and resection of the affected bowel segment. The decision to perform a partial caecectomy, rather than an isolated appendectomy, was guided by intraoperative findings and aimed at ensuring complete removal of the pathological lead point of intussusception. The patient had an uneventful recovery, highlighting the safety and efficacy of laparoscopic intervention in such cases.

## Conclusions

Overall, this case highlights that appendiceal endometriosis is a rare histopathological diagnosis that poses a significant preoperative diagnostic challenge. Endometriosis should be considered even beyond reproductive age, particularly with atypical presentations, as seen in our patient. A history of menstrual irregularities should heighten clinical suspicion for this condition. CT scans and colonoscopy are vital in preoperative assessments, but correctly identifying the underlying condition can be difficult. This case also highlights the importance of multidisciplinary team discussions for complex cases. Interestingly, it is difficult to attribute the cause of her symptoms to appendiceal endometriosis, or merely an incidental finding from her workup for gastritis. Nonetheless, the laparoscopic approach has shown both safety and effectiveness in diagnosis and treatment. Prompt surgical intervention is crucial for resolving intussusception and treating appendicitis to prevent serious complications.
